# Comparison of Traditional and Novel Drying Techniques and Its Effect on Quality of Fruits, Vegetables and Aromatic Herbs

**DOI:** 10.3390/foods9091261

**Published:** 2020-09-09

**Authors:** Ángel Calín-Sánchez, Leontina Lipan, Marina Cano-Lamadrid, Abdolreza Kharaghani, Klaudia Masztalerz, Ángel A. Carbonell-Barrachina, Adam Figiel

**Affiliations:** 1 Agrofood Technology Department, Universidad Miguel Hernández de Elche, 03312 Orihuela, Spain; leontina.lipan@goumh.umh.es (L.L.); marina.cano.umh@gmail.com (M.C.-L.); angel.carbonell@umh.es (Á.A.C.-B.); 2 Thermal Process Engineering, Otto von Guericke University, P.O. 4120, 39016 Magdeburg, Germany; abdolreza.kharaghani@ovgu.de; 3 Institute of Agricultural Engineering, Wrocław University of Environmental and Life Sciences, P.O. 37/41, 51-630 Wrocław, Poland; klaudia.masztalerz@upwr.edu.pl (K.M.); adam.figiel@upwr.edu.pl (A.F.)

**Keywords:** drying trends, drying techniques, dehydration, combined drying, food properties

## Abstract

Drying is known as the best method to preserve fruits, vegetables, and herbs, decreasing not only the raw material volume but also its weight. This results in cheaper transportation and increments the product shelf life, limiting the food waste. Drying involves the application of energy in order to vaporize and mobilize the moisture content within the porous products. During this process, the heat and mass transfer occurs simultaneously. The quality of dehydrated fruits, vegetables, and aromatic herbs is a key problem closely related to the development and optimization of novel drying techniques. This review reports the weaknesses of common drying methods applied for fruits, vegetables, and aromatic herbs and the possible options to improve the quality of dried products using different drying techniques or their combination. The quality parameters under study include color, bulk density, porosity, shrinkage, phytochemicals, antioxidant capacity, sugars, proteins, volatile compounds, and sensory attributes. In general, drying leads to reduction in all studied parameters. However, the behavior of each plant material is different. On the whole, the optimal drying technique is different for each of the materials studied and specific conditions must be recommended after a proper evaluation of the drying protocols. However, a novel or combined technique must assure a high quality of dried products. Furthermore, the term quality must englobe the energy efficiency and the environmental impact leading to production of sustainable dried products.

## 1. Introduction

Drying is an ancient and unparalleled physical procedure of food conservation used for direct preparation of food products as well as for further processing in the food industry. It has always been a valuable and common practice of conservation, ensuring the availability of food and medicinal products all year long. Drying used to be natural and simple as the process was driven by solar energy. Nowadays, it became more sophisticated and complex as it uses a lot of equipment and the drying parameters are carefully examined and optimized at every stage of the process. Emerging new methods have been extensively studied in terms of chemical and biochemical changes in the product during the dehydration process. Drying not only preserves the product but also can have a positive impact on materials quality e.g., in spices, medicinal plants, herbs, bioactive enzymes, and nuts that can generate value-added compounds during drying [1,2]. 

Drying is the process of water removal, usually driven by heat, from solid and liquid products resulting in solid-dried products. Within a fresh foodstuff exist two types of moisture, firstly the bound moisture characterized by the liquid retained in the microstructure of the solid part and secondly the unbounded moisture represented by the excess of the bounded water. The bound moisture is represented by a liquid solution retained into a solid matrix. This leads to the coexistence of complex processes during the thermal drying of fresh food products. First, energy is transferred from the hot drying agent to the fresh product. Secondly, an evaporation of the unbounded moisture (free water) occurs, and eventually, water particles bounded within the cellular structure, subjected to diffusion and thus migration, are transferred to the surface of the product, where the water is finally evaporated [3]. Removing the moisture from the fresh product inhibits the bacteria growth and its proliferation increasing the product shelf-life. Moreover, the enzymatic activity, sensory properties, and microbial growth are also affected by the drying process [4]. As explained above, drying mechanism consists of unbound moisture removal (constant rate period) followed by the internal moisture elimination (falling rate period). Even though the surface evaporation occurs, it is crucial to vaporize also bounded water, as only after falling rate period the process results in secure, dried product [5]. 

Drying is known as the best method to preserve fruits, vegetables, and herbs, decreasing their volume and weight thus, reducing the packaging, storage, and transportation costs. Moreover, flavor and texture properties are modified, obtaining a new generation of products such as snacks that can be a healthier alternative to other commercial products such as sweets [6]. Besides, water removal prevents microorganism evolution and harmful chemical reactions and leads to longer storage time [7]. This is often achieved when the water activity is smaller than 0.3. Thus, depending on the method of water removal, there are different types of drying processes such as (i) thermal drying which is divided into air drying, low air environment drying and modified atmosphere drying; (ii) osmotic dehydration in which a solution is used in order to remove the water and finally (iii) mechanical dewatering which uses physical force for drying [1]. 

The most important objectives of drying are: (i) preservation of fresh products, making them available whole year (ii) conversion of the fresh product into a dry one while maintaining or improving its final quality; (iii) reduction of the volume and weight of the product for an easier transportation and storage; and last but not least (iv) sustainable processing as the most popular drying methods use enormous quantities of energy at low efficiency [8]. Thus, the new drying techniques should provide advantages such as higher energy efficiency, better product quality, cost reduction, and lower environmental impact. 

The energy efficiency, food quality, and drying time are the main parameters to be optimized within the ongoing studies. All these drying techniques aim to develop food products with various characteristics. Although plenty of drying machines (around 50 different types of dryers) have already been designed, proved, and used, not all are suitable to be used in the food industry [9]. 

The most popular techniques used for moisture removal in fruits, vegetables, and herbs are briefly summarized in this paper. Also, new methods that emerged by modification of existing ones or by using known techniques previously unapplied in the food drying are described below. This review reports the weaknesses of common drying methods applied for fruits, vegetables, and aromatic herbs and the possible options to improve the quality of dried products using different drying techniques or their combination. A detailed evaluation of selected methods in terms of its effect on physiochemical, functional, and sensory properties of the dried product is also provided. 

## 2. Drying Techniques in Fruits, Vegetable, and Herbs Preservation

**Figure 1:**
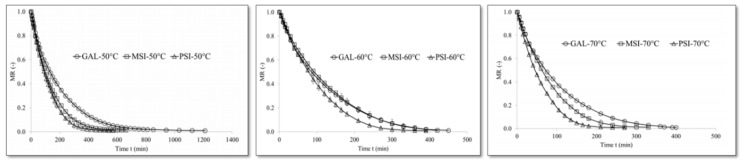
Convective drying of different jujube cultivars at different temperatures [11].

Numerous drying techniques have been developed and used for dehydration of vegetal products over the years. In this section, the most relevant drying techniques, such as convective drying (CD), spray drying (SD), freeze-drying (FD), and osmotic dehydration (OD) are reviewed and their characteristics provided (Table 1). However, these broadly applied methods are not without some drawbacks and therefore extensive research has been carried out to limit these negative aspects as well as minimize the energy consumption throughout the process (Figure 1). According to other authors [10] novel drying technologies that might be accepted by the food industry include energy saving solutions such as dryers with the use of heat pumps, combination of existing technologies in order to optimize the cost and quality of dried products, and every method that allows better control over process conditions and food quality. Hence, these new technologies are described and compared with existing, conventional methods in this section. 

### 2.1. Heat Pump Drying (HP)

Energy losses during the conventional hot air drying are quite significant. Therefore, many methods have been designed to focus on recovering of the exhausted air in the process [12]. However, those methods could only recover sensible heat from the exhaust losing the rest of the heat in form of the latent heat of steam. To avoid this phenomenon, heat pump dryer was designed. In this type of dryer, a refrigerator is used in order to recover the latent heat by water condensation. In fact, this dryer is an improved convection dryer with refrigeration system, which contributes to energy efficiency and improves the product quality limiting negative environmental impact. In the process, dry heated air is supplied to the product as a result releasing humid air. The air travels to the heat pump evaporator, where it is condensed, allowing the latent heat of vaporization to be reused for reheating of the drying air. The advantage of this drying method is the reduction of time and temperature due to the relative humidity decline when compared to the conventional hot air dryer [1,13]. 

An alternative to the compression heat pump is a chemical heat pump. This method is considered as one of the most energy efficient and consists of a solar collector, storage tank, and a drying chamber. The chemical heat pump stores misused heat from the dryer exhaust or solar energy in the form of chemical energy and release it at different temperatures during the drying process. This method works by utilizing the reversible chemical reaction needed to change the temperature level of the thermal energy stored by the chemical substances. The chemical substances such as metal hydrides are very important in absorbing and eliminating heat. This method has the advantages of reducing energy consumption and is designed for a continuous operation [13]. 

### 2.2. Electromagnetic Radiation Techniques

Many conventional drying methods use hot air obtained through electric heater or flue gas to enforce heat transfer between the hot air and the material principally through convection. However, there are plenty of other methods that use electromagnetic wavelength spectrum as energy. Electromagnetic waves of a certain length reach out to the product generating in this way heat, which increases the drying rate [1]. This method works by indirect electro heating because the electrical energy is first converted to electromagnetic radiation to later be transformed into heat in the food product [14]. Some of the drying techniques using this mechanism are described below: 

#### 2.2.1. Microwave Drying (MD)

Microwave drying is based on the volumetric heating occurring when electromagnetic waves pass through the material causing a molecules oscillation. This oscillation generates thermal energy that is then used to remove water from the wet material. Microwave radiation is included in the electromagnetic spectrum, and its wavelengths range from 1 mm to 1 m. The most used frequencies within the food drying are 915 and 2450 MHz. This drying method is able to obtain high quality dried products with reduced costs and high energy efficiency due to the volumetric heating that is spread through the whole sample reducing the time of drying when compared to the conventional methods (hot air-based ones e.g., convective drying described in Table 1). However, it is considered to cause product damage due to improper heat control and mass transfer during the process [15]. Hence, researchers recommend combining this technique with other techniques e.g., by combining the use of microwaves with reduced pressure (microwave-vacuum drying described in the next section). 

#### 2.2.2. Infrared Drying (ID)

The infrared drying occurs by the exposure of the fresh product to electromagnetic radiation in the wavelength range of 0.8–1000 µm. The infrared radiation energy is transferred from the heating source to the product surface. However, the surrounded air is not heated in the process. This method is one of the most appropriate to be used in combination with conventional drying methods due to the equipment simplicity and energy savings. In addition, it is considered to produce a quick and efficient heat transfer obtaining in this way a better organoleptic and nutritional value of the product with a uniform heating and lower final costs [16]. 

#### 2.2.3. Radio Frequency Drying (FR)

This technology can be used not only for wireless communication but also in food processing. Radio frequency heating consists of the interaction between electromagnetic field, which is produced by radio frequency generator, and the molecular species in the product. Thus, the food product is situated between two electrodes displayed to an electric field which alternates around 40,000,000 times per second. The electric fields alternate and so do the polar molecules from the food product creating friction, which heats the whole product. As the water is naturally bipolar it gets heated leading to the evaporation in the process [5]. Radiofrequency has been widely studied as an alternative to conventional hot air-drying process (convective drying) in different horticultural products such as apple slices and snack foods [14]. 

#### 2.2.4. Refractance Window Drying (RW)

This drying technique includes three types of heat transfer mechanisms, (i) convection, (ii) conduction and (iii) radiation. All these heat transfer modes are needed in order to obtain an energy efficient drying method. The product submitted at refractance window must be of liquid or semiliquid texture. The material is applied to a conveyer belt surface, normally an infrared transparent plastic which floats on the area of heated circulating water. This method works by refractive principle of the water surface that creates a window when infrared energy crosses by. The infrared window is formed at the contact between the wet material and the transparent plastic and permits direct infrared energy transfer to the material. Studies on pure pumpkin concluded that the drying time for this method is very short [17]. Refractance window dehydration is conducted under atmospheric pressure and lower temperatures (~30 °C), being a good option for heat-sensitive foods. This method has emerged as a new low-cost possibility for dehydration of vegetal material such as mango, avocado, and herbs [18]. 

### 2.3. Explosion Puffing Drying (EPD)

This method is usually applied in an intermediate phase of the drying process and is caused by the product bounded water vaporization and its expansion due to an abrupt pressure decrease or temperature increase. In this moment, the released vapor is used for both development of an internal structure or expansion and/or breaking of an existing one by producing a porous structure, saving time and energy. There are different methods of puffing, such as high-temperature and short-time air puffing and superheated steam puffing. Explosion puffing drying (EPD) system consists of a puffing chamber, vacuum chamber, vacuum pump, decompression valve steam generator, and air compressor. This method is usually combined with CD and FD and is used as a cheaper alternative to FD products [19,20]. 

### 2.4. Low-Pressure Superheated Steam Drying (LPSSD)

The benefits of low-pressure superheated steam drying (LPSSD) results from the reduction of operation temperature due to lowered pressure and completely lack of oxygen as the drying agent is a steam instead of hot air, which is commonly used for heat and mass exchange in traditional drying methods. The process of dehydration using superheated steam takes place in insulated drying chamber under reduced pressure maintained by vacuum pump. A steam trap is installed to reduce the excess steam condensation in the reservoir, which receives the drying agent from the boiler. With the use of a heater equipped with the temperature control system the initial steam condensation during the start-up period is reduced considerably. A variable-speed electric fan is used to disperse steam throughout the drying chamber. 

### 2.5. Combined Drying Methods

Combined drying methods represent the next group of novel drying techniques that overcome the shortcomings mentioned before by combining the advantages of selected methods and reducing negative aspects occurring when only one technique is applied. There are several combined drying methods. However, some of them deserve special attention due to their applicability in the food industry. 

#### 2.5.1. Microwave-Assisted Convective Drying (CD-MD)

Hot air is an effective drying medium to produce dried fruits, vegetables, and aromatic herbs. However, as presented in the Table 1. Convective drying has some weaknesses i.e., long time of drying and the crust formation on the product surface due to the high temperatures. These issues can be diminished if microwave-assisted convective drying is applied. Therefore, the hot air reduces the products surface unbound moisture while the microwave energy eliminates the bound moisture from the inside of the product via volumetric heating. Nevertheless, there is still the need for further development regarding the time at which microwaves should be optimally incorporated into the process, whether when the drying rate starts to fall or when the drying rate is already falling or maybe even at very low moisture content [21]. 

#### 2.5.2. Vacuum-Microwave Drying (VMD)

Vacuum-microwave drying is a modern technique that might overcome the conventional drying weaknesses having an ability to enhance the quality of the dried products. In general, vacuum-microwave drying (VMD) gathers the four most important requirements for food drying: high operational speed, high energetic efficiency, low operational costs, and high quality of the dried product. The process involves the use of vacuum, which assures a quick mass transfer and low temperature and is combined with the microwave heating which guarantees an accelerated energy transfer. Thus, all together result in prompt, low temperature drying process. In addition, the absence of air prevents the product oxidation. This system is not yet very common in the food industry; however, there is a large number of scientific studies that have successfully applied this method to obtain better quality products (including nutritional and sensory properties), such as fruits, vegetables, and aromatic herbs. However, one of its major limitations is the non-uniformity of the microwave radiation, which induces over-heating in borders and corners of the sample [13,22,23,24]. In recent years, several studies have been focused on using VMD, because of the shorter drying time and lower temperature in comparison with microwave drying. Several vegetal materials have been studied under this technique such as apple, blackcurrant, blueberry, pomegranate, garlic, strawberries, cranberries, and tomatoes [2,23,25,26,27]. 

#### 2.5.3. Convective Drying Followed by Vacuum Microwave Drying (CD-VMD)

**Figure 2:**
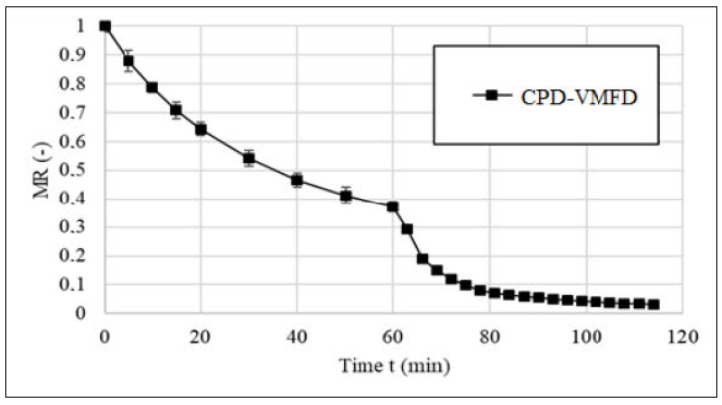
Combined drying consisting on convective pre-drying (CPD) at 60 °C and vacuum microwave finishing drying (VMFD) at 360 W of hemp [30].

Combining convective drying with vacuum-microwave drying leads to obtaining products with improved quality with lower cost of the process as well as lower energy consumption. The process consists of two stages: in the first stage, the fresh product is subjected to a convective pre-drying followed by the second stage in which a vacuum microwave finishing drying is applied to the product. The convective pre-drying diminishes considerably the unbound moisture of the fresh material without affecting their bioactive compounds. Later the vacuum-microwave finishing drying brings the moisture content to the desired level. These two combined drying processes have been reported to be more effective than either of the methods applied separately. The positive impact on the quality was observed in large number of fruits and herbs: sour cherries, jujube, orange peel, beetroot, blackcurrant, pumpkin, plums, and hemp [2,28,29,30] (Figure 2). 

#### 2.5.4. Fluidized Bed Drying (FBD)-Assisted by Microwaves, Far Infrared Rays, and Ultrasounds

Traditional fluidized bed drying can be assisted by microwave energy in a similar way to convective drying. However, this method requires different steps of drying and especially further research on the usefulness for various products. Also, the initial system costs are immense. 

Far infrared rays are found at the farthest side of the visible rays and have the purpose to raise the temperature of the food product to the above wavelength, which coincides with the vibration of the molecules. As a difference to the microwave-assisted fluidized bed drier this method can be applied in any stage to control the matter components quality. For instance, it was possible to control the influence of allicin (the organosulfur compound of garlic) in the drying process when far infrared rays assisted fluidized bed was applied in the first stage of drying. In addition, functional components such as amino acids were not affected by both individual fluidized bed, or combined with far infrared rays methods. 

It is considered that high power ultrasound application to the heat-sensitive horticultural products can raise drying rate by accelerating the mass transfer process contributing to a high quality of dried product. These important aspects can be achieved due to the lower temperatures and times needed for the drying treatment. It was found that using ultrasound methods for fruits and vegetables could avoid negative effects such as shrinkage, color darkening, cracking, or nutritional changes due to the low temperatures needed within this method. Besides, it is considered not only to have an intuitive mechanism of utilization but also to be low-cost and energy efficient method [31]. 

#### 2.5.5. Intermittent Drying (IMD) of Food Products Assisted by Temperature, Pressure, Humidity, Convection, Radiation, and Microwave

As previously mentioned, drying is probably the most energy process of the major industrial process. Intermittent drying has been considered as one of the most energy efficient drying processes. Intermittent drying is a drying method where drying conditions are changed with time. It can be achieved by varying drying air temperature, humidity, pressure or even mode of heat input [32]. Intermittent drying can be accomplished by controlling the supply of thermal energy, which can be achieved by varying the airflow rate, air temperature, humidity, or operating pressure. One can also vary the mode of energy input (e.g., convection, conduction, radiation, or microwave) to achieve intermittency [32]. 

Regarding the food quality, authors reported that the intermittent drying can reduce the browning effects, the hydro-thermo-mechanical stress inside sample and the chemical reactions which help to protect the bioactive compounds of the product [33]. Intermittent drying is a technique specially developed to overcome essential limitations of convective drying which are longer time and energy consumption, case hardening and low-quality products [33]. Thus, is an effective method developed to improve the drying kinetics, enhancing product quality, and reducing energy consumption. 

Intermittent microwave convective drying (IMCD) significantly reduces drying time and improves product quality compared to convection drying and overcomes the problem of overheating that persist in continuous microwave convective drying. Moreover, the non-uniformity of temperature distribution is one of the major drawbacks of microwave drying which can be minimized by supplying microwave power intermittently [34]. 

This method (IMCD), it has been already used in drying of thermolabile plant-based food products due to its soft processing conditions which protect the sample from overheating and product deterioration [33]. For instance, during the drying period the superficial moisture is evaporated, and the inner moisture is carried to the surface; this repeated process of rewetting help to reduce the overheating maintaining stable thermolabile product characteristics such as color, pigments, browning, etc. Moreover, the reduction in oxidation, hydrolytic enzymes, and microorganism can be also reduced due to the short time of heat during IMD which can inactivate them without damaging the heat-sensitive bioactive compounds and enhance the product shelf life [33]. 

Authors reported that using the optimum level microwave power and intermittency could significantly enhance the preservation of nutrient contents, microstructure, and color of the dried sample. For instance, using IMCD at 1:4 power ratio in kiwifruit, was the optimum condition with the highest ascorbic acid retention, the lowest color change and with a porous structure resembling the fresh sample; however, using higher microwave density a (1:3) the highest polyphenol content was maintained [35]. 

Finally, a study on the effect of IMCD on a heat-sensitive fruit such as Red Flesh papaya (cv. Red Hill) was also reported [36]. The authors developed an IMCD model describing simultaneous heat and mass transfers, together with microwave volumetric heating (for temperature of 60 °C, 100 W and 1:3, 1:4, and 1:5 power ratios) to predict the distribution of moisture and temperature and its effect product quality. They concluded that power rate had a key role in quality attributes as was also above-mentioned. For instance, around 70% of the ascorbic acid was degraded using 1:3 power rate, but these losses were decreased when the power rate was reduced to 1:4 or 1:5. Additionally, the total phenolic content, was reported to be degraded significantly during the early stages (first 60 min) but was stabilized at later stages. 

## 3. Product Quality Parameters Affected by Drying Methods

Food drying usually results in product deterioration not only from a sensorial point of view but also from a physicochemical and nutritional one. As explained in the previous sections, the conventional methods of drying are more susceptible to physical and chemical degradation in the final product. For this reason, it is essential to use an appropriate drying method for each product and select the adequate conditions that will reduce possible changes to a minimum. In the following section, some of the physical parameters influenced by the drying methods are described. Moreover, Table 1 and Table 2 summarizes all the drying methods and their effect on quality parameters discussed in this section. 

**Table 1:** Characteristics of selected conventional drying methods.

Drying Method	Drying Agent	Feed Type	Mechanism	Advantages	Disadvantages	Application	References
Convective drying (CD)	hot drying air	Solids—fruits, vegetables, fruit and vegetable pomace	Moisture exchange between the food product and the hot air flowing through the drying chamber	Long shelf-life, simple design; Easy operation; Low cost	High inlet gas temperature or very dry gas; Long drying time, exposure to oxidation; Generates off flavors; Crust formation on the product surface due to the high temperatures	Food industry; Vegetable and fruit dry products; Pomace processing—functional ingredients production	[31,37,38,39,40]
Spray drying (SD)	hot drying gas (usually air)	Liquid—i.e., juices, purée, solutions, vegetable milk	Transformation of liquid product into dry powder form in one-step processing operation	Low moisture content and high-quality products; Long shelf-life; Similar size and shape of dried material; Continuous operation Lower cost than freeze-drying	Might lead to bioactive compounds loss and stickiness due to the high temperature, equipment size, products with large fat content require a defat process, high installation cost	Powder production; Microencapsulation; Production of instant powders	[41,42,43,44]
Freeze-drying (FD)		All types of food	Two steps process: (1) freezing the water from the raw material; (2) heating of the frozen solid to induce the moisture sublimation	Prevents oxidation damages; Minimize chemical compounds changes; Minimal shrinkage and shift of soluble solids; Retention of volatile compounds; Maintenance of porous structure	Very high facilities cost; Slow and expensive process	Production of heat-sensitive compounds i.e., vitamins, microbial cultures, and antibiotics; Production of high-quality products with high final cost: exotic fruits, vegetables, soup ingredients, mushrooms, and juices	[1,2]
Osmotic dehydration (OD)	sugar, salt (sodium chloride) solutions, concentrate juices, polyols solutions	Fruits, vegetables	Moisture reduction by immersion of the raw material in a high osmotic pressure solution → moisture transfer from the food to the solution driven by the difference in osmotic pressure	Maintenance of the physicochemical and sensory parameters; When carried out in concentrated juices might enhance product quality	High final moisture content; Usually needs further drying; High content of sugar or salt in the product when dehydrated in this type of solution; Difficulty in predicting final chemical composition when dehydrated in concentrated juices	Fruit chips production; Production of dried fruits i.e., plums as a pre-treatment before further drying	[45,46,47,48]
Intermittent drying	hot air, microwave power, vacuum and infrared	Fruits, vegetables	Intermittent microwave heating is led by applying microwave energy as sequential pulses, where power ratio has an important role in drying kinetics	Protect bioactive compounds, color, texture; reduce the browning effects and enhance the shelf life.	Higher power ratio can damage important compounds such as ascorbic acid.	Plant-based food material; Fruits: kiwi, papaya, banana, guava, carrot, etc.	[33,35,36]

**Table 2:** Effects of drying methods on the quality of dried materials.

Drying Method	Color	Structural Properties	Polyphenols Content	Antioxidant Activity	Volatile Compounds	Essential Oil (EO) Content	Sensory
Convective drying (CD)	color changes, generally darkening of the product (blueberries, black mulberries) improved color in case of blackcurrant powder	product hardening, high shrinkage, dense structure, low porosity, high bulk density; when combined with ultrasounds-higher capacity of dehydration in mushrooms, Brussel sprouts, cauliflowers	reduction of TPC in i.e., chokecherries, chokeberry, chokeberries, moringa leaves, and mango cubes	high reduction of antioxidant activity in many products (chokecherries, blueberries, chokeberries, mango cubes)	generally high loss of volatiles; higher content than for other methods on the studies on shitake mushrooms and chanterelle	higher yield of essential oil than during MD of herbs (rosemary and basil)	generate off flavors, decrease of fresh, floral, herbaceous attributes
Microwave drying (MD)	better preservation of color than CD	high porous materials in the studies in potato and carrots decreased porosity in the studies on apple and banana	retention of polyphenols in moringa leaves	retention of antioxidants in moringa leaves	high loss of volatiles, but lower than CD	better yield and preservation in basil and coriander	-
Vacuum drying (VD)	-	high porosity in the studies on apple and banana low porosity on the studies of potato and carrot	retention of polyphenols in moringa leaves	retention of antioxidants in moringa leaves	-	-	-
Vacuum-microwave drying (VMD)	Improved color in case of blackcurrant powder	low shrinkage in comparison to CD but higher than FD, porous structure, better than CD in the studies on chokeberries, faster reconstitution, lower bulk density than CD	higher than CD in the studies on sour cherries	higher than CD in the studies on sour cherries	higher loss of some compounds than CD	increased EO yield in garlic, higher loss of EO than CD of rosemary	decrease of fresh, floral, herbaceous attributes, increase of sweetness, bitterness and adhesiveness
combined convective drying followed by vacuum-microwave drying (CD-VMD)	Slight degradation of color (better than CD and VMD); Improved color in case of blackcurrant powder	lower bulk density than CD	higher than CD and VMD in the studies on sour cherries, chokeberries,	higher than CD and VMD in the studies on sour cherries, Saskatoon berries, chokeberries	higher retention in the studies on chanterelle than other drying methods	increased EO yield in thyme, oregano, and rosemary	-
Freeze-drying (FD)	good preservation of natural color in many studies (i.e., black mulberries)	no shrinkage, no collapse, highest porosity, loss of elasticity, viscous material, lower bulk density than CD	preservation of TPC (black mulberry, chokeberries)	preservation of antioxidants	major loss in drying of parsley, low loss of flavor and aroma	preservation of most EOs	-
Osmotic dehydration (OD)	good preservation of color, change of color due to the osmotic solution properties (when concentrated juice is used as osmotic solution)	when combined with FD—strengthen the material structure when used as a pre-treatment before CD or CD-VMD—increase porosity lower bulk density than CD	increase when dehydrated in chokeberry, sour cherry solution, degradation in the studies on sour cherries	degradation in the studies on sour cherries increase when carried out in concentrated pomegranate and chokeberry juices	-	-	-
Heat pump drying (HP)	improved color in rosemary and parsley brown areas when applied on nuts	good preservation of the structure in some herbs than other drying methods	good preservation of polyphenols in drying of herbs	good preservation of polyphenols in drying of herbs	volatiles retention on the studies on ginger	-	-
Fluidized bed drying (FB)	good color retention	-	no significant reduction in kafir leaves	no significant reduction in kafir leaves	-	-	-
Refractance window drying (RW)	decreased browning reaction in pomegranate leather	positively affected	retention of polyphenols high content in pestil pomegranate	retention or improved antioxidant activity in the studies on asparagus, sweet corn and tomatoes high content in pestil pomegranate	-	-	-
Intermittent drying	reduce the color degradation	maintain the product microstructure obtaining a porous structure similar to the fresh sample	Retention of polyphenols	retention of ascorbic acid, carotenoids, and so increasing in the antioxidant activity	retains the lower volatile compounds (due to microwave energy penetration which accelerates the disruption of the cell membranes that ultimately releases the volatile compounds faster)	-	protect cells from oxidative injury, providing better sensory quality

### 3.1. Color Changes during Dehydration

Maintaining of natural color in dried food products is very important as the visual appearance is one of the first judgments made by consumers. Color together with size, gloss, shape, etc. form the appearance and represent a valuable indicative parameter used in quality control. In the food industry, color modification has enforced enhancing of the visual appearance by adding coloring agents, stimulating high consumer acceptance in foods, particularly dried fruits, and vegetables. One of the most challenging aspects for drying methods is to perform the process in a way that will result in an attractive color for the final product. 

Most of the time color variations during drying are related to browning reactions which can be caused both by enzymatic and non-enzymatic reactions. The enzymatic browning occurs in fruits and vegetables due to the phenolic compounds oxidation by polyphenols oxidase, which starts the generation of the brown pigments (melanins) called *o*-quinones. However, there are alternatives to prevent this degradation such as the previous inhibition of enzymatic system by using sulphites [39]. On the other hand, the non-enzymatic browning is produced by different types of non-enzymatic reactions such as Maillard, caramelization, and ascorbic acid oxidation, and is influenced by water activity, temperature, pH, and product composition. Browning is accelerated when the level of water content is intermediate and decreases at very low or very high levels of moisture content. Consequently, browning is harsh close to the end of drying process due to the low levels of moisture remaining in the product. In carbohydrate dried foods (especially fruits and vegetables), changes of color after dehydration are linked with the presence of high amount of reducing sugars such as glucose and fructose. These compounds interact with amino groups from proteins and undergo Maillard reaction during the exposition to air at high temperatures, long drying times and the amount of water. In addition, color changes appear due the degradation of some thermosensitive compounds such as anthocyanins and carotenes, which results in a loss of functionality and color. A quick dehydration of the product to 15–20% moisture content can reduce Maillard reaction time. For this reason, the drying methods are designed to accelerate the drying time in order to achieve the desired moisture content while reducing the browning time [49,50]. 

Since the color can be affected by several factors (raw material properties, drying methods, additives), it is important to discuss the changes with the total color differences (*ΔE*). Colorimeter is used in order to determine the color of the product by measuring CIE L*, a*, b* color coordinates values for each sample. To calculate *ΔE* authors used the following equation:

(1) $$\Delta E=\;{\left[{\left(L-L^{*}\right)}^{2}+{\left(a-a^{*}\right)}^{2}+{\left(b-b^{*}\right)}^{2}\right]}^{0.5}$$

In which *L*, *a*, *b*, represents the blank sample while the L*, a*, b* are the values obtained for the dried samples. The *ΔE* indicates the degree of total color change while the L*, a* and b* coordinates refer to: L* (whiteness-darkness), a* (red-green) and b* (yellow-blue) values. Authors have found that if the *ΔE* is between 0 and 1.0, the color difference cannot be distinguished from a sensorial point of view [51,52]. 

Degradation of product original color appears at long drying time exposure and high temperatures. According to many studies in this area conventional techniques, such as convective drying, are considered to cause significant losses in color, while the novel technologies based on combined drying methods (i.e., convective and microwave techniques) are limiting the greatest color alterations by minimal heat exposure or by using high temperatures combined with short time and pH adjustment. 

Color changes by different drying methods have been already demonstrated for numerous plant materials (Table 3), i.e., blackcurrant [53], pomegranate [54], soya [55], *Piper borbonense* [56], sour cherries [57], blueberry [27], black mulberry [20], and, chokeberry [58]. 

In the studies on basil leaves microwave drying, convective drying and freeze-drying were compared. Microwave drying prove to be the best method in terms of color retention as it led to fewer changes than convective drying [59]. 

Wojdyło et al. [52] studied the bioactivity of sour cherry dried by three methods of drying: freeze-drying, convective drying and vacuum-microwave drying. The authors confirmed that using low drying temperatures, short time, and narrow exposure of the raw material to the oxygen present in air are essential to maintain the bioactivity of the product. For this reason, the VMD was found to be recommended in order to assure the abovementioned conditions rather than the CD as this method resulted in similar results to the FD method which was considered as the control. The best parameters in this study were VMD at 480 W until 1 kg/kg dm moisture content followed by drying at 120 W until the end. These parameters assured an attractive final product color linked to a proper anthocyanin content along with a higher polyphenols content and antioxidant capacity [52]. Moreover, Šumic et al. [60] worked on the vacuum-drying process optimization for frozen sour cherries and concluded that the higher product quality can be obtained if it is performed at temperature of 54.03 °C together with a vacuum pressure of 148.16 mbars. The quality in this study was described by the maximum antioxidant activity in dried cherries along with the maximum amount of total phenolic content, Vitamin C and anthocyanins assuring in this way the minimal color change and the desired sample texture [60]. Finally, Horuz et al. [57] also studied sour cherry drying subjected to both convective drying (50 °C, 60 °C, 70 °C) and combined convective-microwave drying (120 W, 150 W, 180 W coupled with CD 50 °C, 60 °C, 70 °C). The authors concluded that the sour cherries color parameters were similar for both CD and CD-MD. However, it should be noted that the drying time was reduced for CD-MD when compared with the CD method. Consequently, CD-MD increased not only the final product quality and its rehydration capacity but also the energy efficiency which is essential for environmental impact and costs [57]. 

Zielinska et al. [27] studied the CD (60 °C and 90 °C), VMD and their combination CD (60 °C and 90 °C)-VMD in blueberry drying process. As explained above, degradation of anthocyanins results in color degradation due to the location of these compounds in the material as they are mainly situated in the blueberry outer layer. The authors have found that the content of anthocyanins was higher if the samples were dried with VMD when compared with CD. However, when comparing all the methods used in this study, the CD at 90 °C combined with VMD was found to be the most suitable drying method regarding the retention of anthocyanin in blueberries and color preservation. Nevertheless, if compared with the fresh frozen blueberries the anthocyanin content was reduced to 30% in dried samples for the combined CD (90 °C)-VMD. This agrees with other authors that had worked with Saskatoon berries dried with combined CD-VMD and showed an anthocyanin content reduction to 38% [27,61]. 

In studies about black mulberries using 4 different drying methods such as CD, FD, CD, combined with EPD and FD combined with EPD, the most suitable drying process after FD was found to be FD-EPD. Black mulberry dried with this method was found to present the best color and so to retain the most anthocyanin content when compared to both CD and CD-EPD. This happened due to the vacuum environment which helped to retain more pigment. On the other hand, the thermal process of CD contributed to pigment degradation resulting in less positive color alterations [20]. 

Samoticha et al. [58] studied drying of chokeberries using different methods. For this reason, FD, vacuum drying (VD), CD, VMD and combined convection drying followed by vacuum-microwave drying methods (CD-VMD) have been used. The authors concluded that FD resulted in maintaining the most of bioactive compounds content in dried fruits when compared with other methods. In addition, the color correlated with the anthocyanin content along with other functional components such as phenolic compounds and antioxidant capacity and was found to deteriorate due to the air temperature increment during CD method as well as the raise in the material temperature for VMD. Furthermore, the combined method CD-VMD (especially with the following parameters: CD at 70 °C for 2 h followed by vacuum-microwave with 360 W reduced to 240 W) was found to result in the highest quality final product when compared to the CD and MVD [58]. 

Furthermore, an osmotic dehydration pre-treatment can improve the color attributes in plant materials as confirmed by Cano-Lamadrid et al. [62] in their study on pomegranate arils. In this study, they have used osmotic dehydration followed by a combined CD-VMD to improve the quality of the *Mollar de Elche* pomegranate arils. They observed that using osmotic dehydration as a pre-treatment depending on the osmotic solution (in this case, the juice obtained from the *Wonderful* variety of pomegranate, which is characterized by an attractive color) considerably increased the dried arils color due to the anthocyanin present in the osmotic solution [62] There are also many studies regarding drying of powders. Authors such as Michalska et al. [53] studied the effect of different drying techniques (FD, CD 50–90 °C, MVD 120–240–360 W, CD-MVD) on color of blackcurrant pomace powder. Within this study, it was observed that the drying process improved the powder color when compared to the raw material. In general, the red components were faster released when using VMD or FD methods due to the lower pressure administered. In addition, chroma value (C*), which represents the degree of saturation and is an important marker of product color intensity as kept by human eye, also showed a color enhancement when drying methods were used. However, the color results of powder obtained using CD, MVD and CD-MVD were similar to those obtained by using FD which means that color parameter of blackcurrant powders is not affected by processing temperatures, even though is recommended to be evaluated for processing time and costs [53]. 

Tontul et al. [54] have studied the color changes in pomegranate leather (pestil) dried by different drying methods: CD at 50 °C, 60 °C or 70 °C, combined CD-MD (90 W, 25 W or 180 W) at 50 °C, 60 °C or 70 °C and RW drying at 90 °C, 95 °C and 98 °C. The authors concluded that the RW technique decreased the browning reaction providing better color to the final product. Besides, the textural properties, as well as the functional ones, were also positively affected by this method of drying [54]. 

In herbs such as rosemary, parsley, etc., the color and aroma were improved when heat pump drying was used, while Iranian Saffron considered one of the most expensive spices is highly recommended to be dried with this method due to its sensitivity to heat. However, some dried nuts presented brown areas at elevated temperatures [1,13]. 

Soy okara is a byproduct resulting from the soymilk and tofu production with a great nutritive value. In order to avoid food waste, the industry uses different conservation methods and one of them is drying. However, consumer acceptance of dried okara is disturbed due to the darker color when yellow one is expected in the final product. For this reason, authors studied the possibility of preserving the desired color by using the CD method at different drying temperatures (50 °C, 60 °C and 70 °C) and processing time. In this study, authors observed that the browning increment was directly proportional to the processing time. Hence, the longer the time of hot air exposure the higher the color degradation. The study recommended using CD at 50 °C as the best option in order to maintain the nutritional quality, even though the L*, a*, and b* values were similar at the end of the process for all temperatures. In addition, they affirmed that when okara was dried in a jet spouted-bed dryer the browning was reduced [55]. 

Drying impact on *Piper borbonese* was also studied and the changes in the color of the dried pepper by using convective drying at different temperatures (60 °C, 75 °C, 100 °C) were reported. Those changes can be controlled by applying different pre-treatments prior to drying such as blanching and sweating which included maintaining of the samples in a climatic chamber at 35 °C and 99% relative humidity for 24 h. Moreover, optimization of drying process including pre-treatments would definitely improve color of dried pepper. For instance, they concluded that correcting the blanching parameters would help to reduce not only the drying time but also the enzymatic browning [56].

**Table 3:** Color changes of some dried fruits and vegetables by drying methods.

Drying Method ^±^	Conditions	Vegetal Material	Parameter Affected	Reference
CD	50–90 °C	Blackcurrant pomace powder	L*, a*, b*, C*	[53]
	50–70 °C	Soya	L*, h	[55]
	60 °C	Piper borbonense	L*, a*, b*	[56]
	50–60 °C	Pepper	L*, a*, b*	[63]
	30–70 °C	Pumpkin and green pepper	C*, h	[64]
	50–70 °C	Sour Cherries	L*, a*, b*	[57]
	50–90 °C	Chokeberry	L*, a*, b*	[58]
	50–70 °C	Pomegranate	L*, a*, b*	[55]
	60 °C	Apples	L*, b*	[65]
VD	240–480 W	Chokeberry	L*, a*, b*	[58]
VMD	240–480 W	Blackcurrant pomace powder	L*, a*, b*, C*	[53]
	2.5, 1.9, and 1.3 W/g	Carrots	L*, a*, b*	[66]
	240–480 W	Chokeberry	L*, a*, b*	[58]
	320–120 W	Apples	L*, b*	[65]
CD-VMD	50–90 °C/480 W	Blackcurrant pomace powder	L*, a*, b*, C*	[53]
	300 W/40 °C	Herbs: basil, lovage, mint, oregano, parsley and rocket	a*, b*	[67]
	120–180 W/50–70 °C	Sour Cherries	L*, a*, b*	[57]
	60 °C/320–120 W	Apples	L*, b*	[65]
	50–70 °C/90–180 W	Pomegranate	L*, a*, b*	[54]
	50–70 °C/360–120 W	Chokeberry	L*, a*, b*	[58]
CD-EPD	CD 70 °C-EP 80 °C-CD 70 °C	Black mulberry	L*, a*, b*	[20]
RWD	90–98 °C	Pomegranate	L*, a*, b*	[54]

^±^ CD: hot air convective drying; VD: vacuum drying; VMD: vacuum microwave drying; CD-VMD: vacuum microwave drying after hot air convective drying; EPD: explosion puffin drying; RWD: reflectance window drying.

### 3.2. Physical Properties of Dried Fruit

#### 3.2.1. Structure

The structure of food materials can be characterized by density (apparent and true), porosity, pore size distribution, specific volume, particle density, and shrinkage, etc. Among these, bulk density, porosity, and shrinkage are the most common structural properties reported in the literature [68]. The physical properties of dried fruits are very important mostly in terms of rehydration characterized by the ability of the dried product to return to its initial features. It is well-known that many dried fruits and vegetables along with other ingredients are used in breakfast, ready-to-eat meals, or soups and therefore a proper rehydration is necessary. Rehydration depends on various factors such as type of pre-treatment, moisture content, processing method, and drying conditions. As water is removed from the matrix during the drying process significant changes in structural properties can be observed. Thus, in order to develop high quality dried products besides retaining its color and other functional properties it is also necessary to assure a proper rehydration. Drying methods influence the density, porosity, and sorption features of the product thus, the election of drying methods is essential in order to produce high quality product both dried and rehydrated. Consequently, reduced hydrophilic properties are developed due to the cellular rupture, which is irreversible and results in an integrity loss and dense structure formed by broken down and shrunken capillaries. All of this obstruct the water absorbance and so the rehydration. During rehydration, the dried product suffers numerous internal structure changes i.e., moisture, porosity, volume etc. changes. Thus, the ability of the dried product to regain the form from before the drying depends mostly on the internal structure of the dried particle and the degree of deterioration during the drying process of water holding chemical components such as proteins or starch. CD with a higher drying rate at the beginning of the drying process may lead to product hardening, which results in lower reconstitution capacity. Studies on asparagus dried with both CD and FD concluded that higher quality (faster reconstitution and smooth texture) was obtained when hot water blanching pre-treatment was combined with FD. Usually, the products dried with CD method have a dense structure and a high shrinkage contrary to those dried with microwave, which are characterized by a porous structure that leads to faster reconstitution. Microwaves facilitate obtaining porous products due to drying mechanism that uses volumetric heating to evaporate bounded water producing high internal vapor pressure. Combined CD with the use of ultrasounds is recommended in order to control the internal structure of the dried product. The ultrasounds have a mechanical effect on the drying product, contribute to higher capacity of rehydration without significant overheating. The rehydration depends on the damage of the matrix cell and the structural rupture occurring within the drying product. For instance, ultrasound-assisted CD helped to improve the rehydration capacity of the dried product when compared with those dried without ultrasound power. In studies on mushrooms, Brussel sprouts or cauliflowers dried at 80 °C the rehydration capacity was improved when ultrasound pre-treatment was applied at low acoustic intensity (0.5 W cm^−2^) in the shortest time (3 min) [69,70]. When VMD is applied the nutrient and sensory (color) properties are preserved during a longer period. With this method, the energy administered is right to the product molecules and then it is spread to the whole food product instead of only surface. Hence, this method also influences the product texture creating in this way a porous product. 

**Figure 3:**
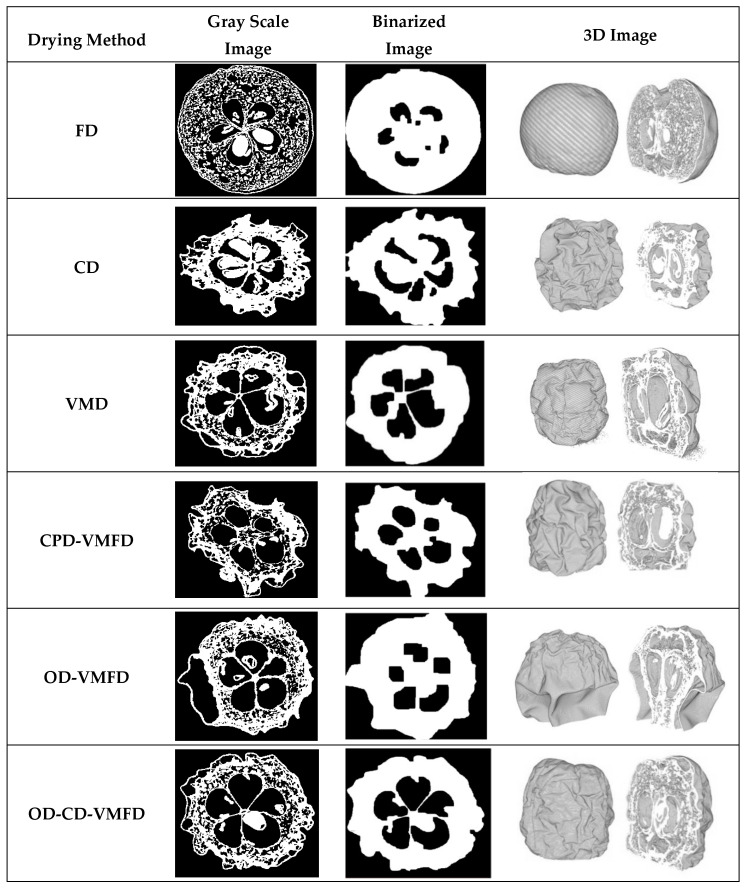
X ray reconstructed images of black chokeberry samples dried using different methods [72].

Usually the internal structure of solids or microstructure of semisolids products is analyzed by using sensory panels or by analytical methods by measuring the physical properties, such as texture with a texture analyzer. However, lately 3D image analysis was found to be helpful in terms of internal structure by giving valuable information on size, volume fraction, wall thickness etc. X-ray microtomography (XMT) is a relatively new technique that provides a non-destructive 3D visualization of the internal structure of objects. In recent years, much attention has been given to expanding this imaging technique to food science in order to help in the study of food microstructure, including fresh and dried products. Recently, XMT has been successfully applied for highly porous products to determine the shrinkage, cracking, and the internal moisture of different products such as dried banana, carrots, chokeberries, apples etc. [71,72,73] (Figure 3). 

#### 3.2.2. Porosity

Porosity is one of the most important features used to describe the texture of dried fruits and to characterize its open structure. Porosity (*ε*) is a measure of empty spaces in the material and it is usually calculated with the apparent density (ρ*_α_*) and true density (ρ*_p_*) of the product by using the equation provided below:

(2) $$\varepsilon =1-\rho_{\alpha }/\rho_{\rho }$$

The apparent density refers to the density of the material, including the pores and it is calculated as the mass of the material divided by its apparent volume while the true density is calculated with the mass of the material divided by its true volume and it refers to the density of the material without the pores. This physical parameter depends on the drying system and a well-suited one would produce materials with a high degree of porosity. Different drying methods have been compared in terms of porosity and FD was found to produce the higher porosity materials (80–90%). However, freeze-dried products were found to have a sensible structure during rehydration, which leads to a loss of elasticity and more viscous material. For this reason, combined techniques are needed in order to reduce this issue. Osmotic pre-treatment combined with FD was found to strengthen the dried product structure. Microwave drying can also produce high porous levels depending on the drying product. For instance, the porosity of microwave dried potato and carrot was around 75%, while for microwave dried apple and banana lower porosity was obtained (60% and 25%, respectively). The same phenomenon was found for vacuum drying technique in which dried banana and apple obtained high porosity (70%), while for vacuum dried carrot and potato porosity values were lower (50% and 25%) [69,72]. 

**Figure 4:**
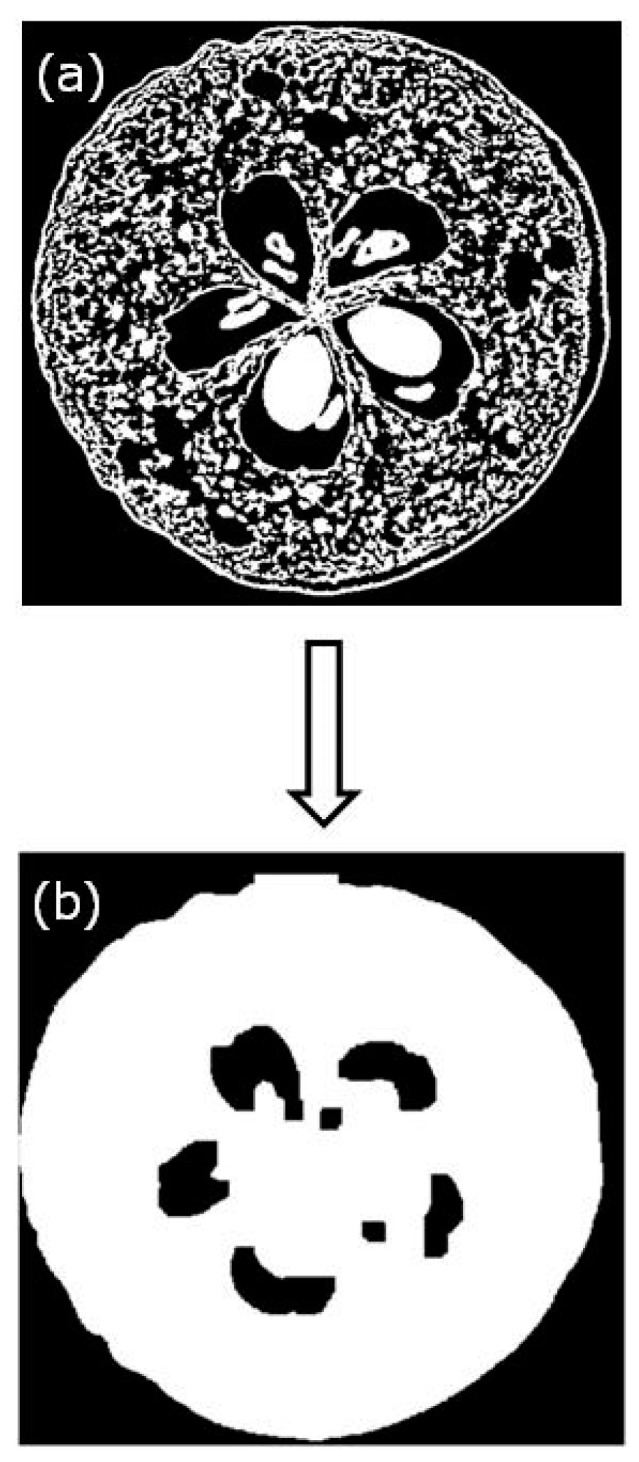
Images used to calculate (**a**) solid volume and (**b**) total volume [72].

Recently, Calín-Sánchez et al. [72] performed a comparison of dried chokeberries dehydrated by several methods. The porosity values oscillated from 18.4 up to 76.3% during convective drying and freeze-drying, respectively. When comparing convective drying with vacuum-microwave drying for the last one, an important increment in porosity (39%) was found. Products in which the osmotic dehydration was used as pre-treatment and combined with convective drying and vacuum-microwave drying have significantly increased their porosity (42.3%) compared to those only dried with CD (18.4%), VMD (38.6%) and combined dried (CD-VMD) chokeberries (28.4%). However, this increment was higher when before VMD an osmotic dehydration pre-treatment was applied leading to values around 45–49% respectively. Consequently, these authors also agreed that freeze-drying achieved higher porosity samples when compared to the other individual drying techniques (CD and VMD). In addition, they have concluded that the other combined techniques (CD-VMD) could enhance these parameters only when osmotic dehydration pre-treatment was applied leading to good results regarding porosity [72]. Porosity as well as the total pore volume were assessed by XMT and reconstructed X-ray images. Porosity and the total pore volume of dried samples were determined from the binarized images; the steps employed for this purpose were as follows: (i) calculation of the solid volume (*V_S_*) from the black and white images and (ii) calculation of the total volume (*V*) after filling the samples without considering the volume of the chokeberry seed (Figure 4). Porosity was calculated using Equation (3) and results were expressed as percentage, and the total pore volume was calculated using Equation (4) and results were reported in mm^3^.

(3) $$\varepsilon =(1-\frac{V_{s}}{V})\times 100$$

(4) $$V_{p}=V_{}-V_{s}$$

Other authors reported that using low-pressure superheated steam drying (LPSSD) helped to improve the porosity degree of the dried products when compared to conventional hot air or vacuum drying. This is possible due to the expansion of the material cells produced by the high-pressure gradient within the product. However, this process is quite slow, so a combined LPSSD with far infrared radiation (FIR) should be applied in order to accelerate the process. Besides process acceleration, FIR also contributes to drying time reduction, increasing the product quality [73]. 

#### 3.2.3. Bulk Density of the Dried Material

Bulk density (*p_b_*) applies to powdered and porous materials, completely occupying the volume of the container (bulk volume) in which they are located. It is defined as the mass of the dried sample (m) divided by its bulk volume (*V_b_*) and is calculated with the following formula:

(5) $$p_{b}=\frac{m}{V_{b}}$$

Low bulk density of the dried products obtained due to the “puffing” effect is mostly desired in order to increase the sensory aspects resulting in higher consumer acceptance. Many factors influence bulk density of the dried products and the most important, besides the porosity that has already been mentioned, is the drying method with its parameters (temperature, microwave power and time). The drying conditions affect not only the apparent volume, which characterizes the individual particles but also the shape of these particles and thus the bulk volume which, in turn depends on the degree of packing of the material. In this way, studies on dried chokeberry showed that both FD and MVD (360 W) were found to be the most efficient methods in terms of bulk density reducing it to around 50% when compared to the CD. In addition, the combined CD-VMD, developed to prevent the sample overheating, was also found to be a good option in order to reduce the bulk density of the dried products up to 55% with respect to the convective dried products. This could be due to volumetric heating occurring when microwaves are applied, which results in puffing of the material. The application of osmotic dehydration improved the values of bulk density when compared to convective dried chokeberries; however, these results were as good as vacuum-microwave dried samples and comparable to freeze-dried samples. Moreover, bulk density has been deeply studied in many other dried fruits; for instance, Yemmireddy et al. [74] studied blueberries (rabbiteye blueberries) and reported that the highest values of bulk density were observed for the samples dried with hot air. Van Arsdel et al. [75] showed that the bulk density of the food product dried to the same moisture content depends on the rate of shrinkage. This statement is strongly affected by the drying method and the drying conditions, such as temperature or time. Longer drying times might have resulted in internal cell destruction and excessive shrinkage of blueberries and hence high bulk density [76]. The shrinkage can also be related to the preservation of foods, the rehydration rate and the biochemical reactions that occur during further storage [72,74,75,77]. 

#### 3.2.4. Shrinkage

Considerable changes in the physical structure of the product, such as reduction in volume and decrease in internal porosity (apparent porosity) can occur during the drying process. Shape and size changes during drying modify not only the dimensions and transport properties of individual particles but also the thickness and porosity of the packed bed in the dryer. The shrinkage results from the reduction of particles porosity, while dehydration increases the bulk density of the material. Experimental data on shrinkage of fruits during hot air-drying reported in previous studies showed products with higher shrinkage and dense structure. Constant porosity and minimal shrinkage are often stated as key assumptions in the model for dryer design. Freeze-dried fruits and vegetables are usually characterized by minimal shrinkage, while the product quality of hot air-dried products is relatively low, mainly due to the hard texture of the products dried by this method [69]. 

Shrinkage of vacuum-microwave dried products is significantly lower, the rate of drying is noticeably higher, and the duration of the drying process is considerably shorter in comparison to convective drying. Puffing phenomenon associated with a fast dehydration is responsible for a porous structure of vacuum-microwave dried products; however, the number of pores is smaller, and the size of pores is bigger when compared to freeze-drying products. Consequently, the shrinkage of vacuum-microwave dried products is slightly higher than freeze-dried ones but significantly lower in comparison to hot air-dried product. Freeze-drying has been found to minimally affect the structure of dried material resulting in no noticeable shrinkage or collapsing in the studies on chokecherries [78]. Combined drying consisting of the osmotic dehydration and the vacuum microwave drying (OD-VMD) might additionally improve the physical properties, e.g., the texture of the dried product. Application of osmotic drying using sucrose reduced the shrinkage and improved the rehydration capacity of vacuum microwave dried pineapple circular discs providing a softer texture and less hardened surface. Torringa et al. [79] reported that the increase in a concentration of the osmotic solution decreased the shrinkage of the mushroom finish dried by combined microwave hot air drying method. This relationship can be explained by increased maximum temperature of vacuum microwave drying at final stage resulting from increased osmotic solution concentration. New techniques ensure a lower shrinkage than traditional methods due to the puffing phenomenon, which is enhanced by a high inner pressure associated with the temperature of the dried material [29,69,79,80]. 

## 4. Effects of Drying on Functional Properties and Nutritional Quality of Food Products

### 4.1. Changes in Phytochemicals Compounds

The presence of phytochemicals, including carotenoids, polyphenols, and vitamins (ascorbic acid), minerals, etc. have been attributed to protective action on degenerative illness. Table 4 shows a recompilation of the main phytochemical compounds (carotenoids, polyphenols, and vitamins) with widely reported antioxidant capacity, affected by drying methods on several plant materials (Table 4). 

The variation of content of these phytochemicals can be affected by temperature, exposure time, levels of oxygen and the presence of light. Several studies indicate that the phytochemicals content in dried products obtained by low temperatures is higher than in dried products obtained by high temperatures [81]. Alternatively, reduction of oxygen levels at the absence of light in microwave, refractance window, low pressure superheated steam, and vacuum drying methods [50] can increase the retention of these compounds. In studies on dried leaves, a higher retention of lipophilic vitamins was found when FD or microwave drying was applied, while the opposite phenomenon happened for the convective dried samples. In addition, when fluidized bed drier (FB) was used to dry kaffir lime leaves, no significant reduction of Vitamin C and A was observed. Regarding the mineral content of moringa dried leaves the temperature had a significant impact on many elements except magnesium and FD was also found to be the most efficient, followed by air drying and oven drying [5]. 

Pomegranate pestil exhibited a high content of phenolic compounds. When combined, drying method was used and it was observed that microwave-assisted drying provides a high content of polyphenols while refractance window ensures a higher anthocyanin and ascorbic acid content in the dried material [54]. Furthermore, carotenoids were found to be less sensitive to time of drying than to temperature while ascorbic acid was reduced within the prolonged drying time. On the other hand, lycopene content was found to be higher when combined drying methods were applied than individual drying. For instance, the tomatoes dried by osmotic-vacuum drying were found to have a higher lycopene content than those dried only with vacuum or convective method. This phenomenon is due to heat and oxygen exposure [81]. 

**Table 4:** Recompilation of different phytochemicals (carotenoids, polyphenols, and vitamins) affected by drying methods.

Drying Method ^±^	Conditions ^±^	Vegetal Material	Phytochemicals	Reference
CD	55–62 °C	Sweet potato	β-carotene Vitamin C	[39]
	50–70 °C	Jujube	Flavonoids Vitamin C	[82]
	65–73 °C	Avocado	Flavonoids Phenolic acids	[83] [40]
	50–70 °C 50–60 °C	Pomegranate Strawberry	Anthocyanins	[84] [85]
	55–65 °C	Cauliflower	Vitamin C	[86]
VMD	120 W–480 W	Jujube	Flavonoids Vitamin C	[40] [82]
CD-VMD	60 °C/480–120 W	Jujube	Flavonoids Vitamin C	[40] [82]
SD	110–130 °C	Tomato pulp	Licopene	[87]
	120 °C	Grapefruit	Flavonoids Phenolic acids	[83]

^±^ CD: hot air convective drying; VMD: vacuum microwave drying; CD-VMD: vacuum microwave drying after hot air convective drying; drying; SD: spray-drying.

### 4.2. Changes in Antioxidant Capacity as a Result of Dehydration

The stability of compounds with antioxidant activity is influenced by many factors, mainly raw material, temperature, and process time. Even though the antioxidants are mostly retained during drying, it is essential to know the retention of the antioxidant capacity for each drying technique in order to choose the right one that leads to high-quality dried products. 

Considerable scientific evidence of the effect on antioxidant capacity and the total polyphenolic content by drying treatments are shown in Table 5. Since many of bioactive compounds degrade during drying, the optimization of the drying process is the key component to obtain the best quality dried product. Studies on antioxidant capacity after drying of asparagus with novel methods such as RW or combined microwave and spouted bed affirmed that the bioactive compounds retention improved when these methods were applied compared to CD. During hot air drying the loss in antioxidant capacity was due to the product exposure to oxygen. In an oxygen-free environment, it is highly recommended to use low temperatures necessary to avoid loss of phenolic compounds and antioxidant capacity due to the thermal and oxidative degradation. This was proved in the studies on mango cubes. On the other hand, RW drying rapidly heats the product which results in a faster release of the phenolic compounds in cell material. In addition, the water loss is severe during the first minute of the operation while the intense vapor pressure produced by the moisture evaporation reduces the partial pressure close to the product and prevents the phenolic compounds from oxidation. This argument was stated by other authors in studies on sweet corn or tomatoes who observed an increment in antioxidant capacity due to the heating process. 

Agreeing that even the natural antioxidants are lost during the process, the antioxidant capacity is enhanced due to the production of new antioxidants. Sehrawat et al. [6] in their study about the production of nutritive dried mango cubes snack found that the low pressure superheated drying method at 70 °C is the most suitable method when compared with vacuum drying and hot air drying, even though this two methods also showed good results at 60 °C [6,88]. Finally, osmotic dehydration pre-treatment in pomegranate and chokeberry juices of pomegranate arils increase not only the antioxidant capacity but also other functional compounds in the studies on pomegranate arils [62]. 

**Table 5:** Recompilation of antioxidant capacity (DPPH, ABTS, and FRAP) and total polyphenolic compounds (TPC) affected by drying methods.

Drying Method ^±^	Conditions ^±^	Vegetal Material	AC Affected	Reference
CD	40 °C	Date fiber	DPPH TPC	[89]
	50–70 °C	Pomegranate	DPPH	[25]
	50–90 °C	Blackcurrant	ABTS TPC	[53]
	50–70 °C	Jujube	ABTS FRAP	[82]
	55–62 °C	Sweet potato	TPC	[39]
	40–60 °C	Cocao bean	TPC	[90]
VMD	240–480 W	Pomegranate	DPPH TPC	[25]
	120 W–480 W	Blackcurrant	ABTS TPC	[53]
	120 W–480 W	Jujube	ABTS FRAP	[82]
CD-VMD	50–90 °C/480 W	Blackcurrant	ABTS TPC	[53]
	50–90 °C/480 W 60 °C/480–120 W	Jujube	ABTS FRAP	[82]
SD	120 °C	Grapefruit	DPPH	[83]

^±^ CD: hot air convective drying; VD: vacuum drying; MVD: vacuum microwave drying; CD-VMD: vacuum microwave drying after hot air convective drying; EPD: explosion puffin drying; RWD: reflectance window drying; SP: spray-drying.

### 4.3. Changes in Nutriotional Quality of Dehydrated Food Products

The reduction of the content of sugars in plant material after drying is due to the browning reactions, especially Maillard reactions. A clear example is given: an increase of temperature and time during convective drying of pomegranate arils caused a reduction of some characteristic sugars (fructose and glucose) [25]. These sugars create conjugates with amino group from proteins. Therefore, the functional properties of proteins can be influenced by drying method, especially, lysine is used as an indicator for protein quality deterioration [83]. A visible example is the change of amino acid profile (mainly, arginine and lysine) of chickpea proteins concentrates among convective drying [84]. As explained in the color section, the Maillard reactions produce changes in the color of the product which results in a darkening and browning usually considered as a negative aspect. However, some authors affirmed that the antioxidant capacity of the product could be improved by Maillard reaction due to the production of melanoidins [88]. 

## 5. Changes in the Volatile Compounds or Essential Oils during Dehydration of Fruits, Vegetables, and Aromatic Herbs

The effect of dehydration on essential oils and/or volatile compounds has been reported mostly in aromatic herbs. The two basic forms of consuming culinary herbs are fresh and dried. Fresh herbs cannot be supplied in a profitable way to all worldwide locations. The essential oil flavor composition of aromatic herbs has been the subject of considerable research in recent years. It is well-known that the presence of essential oils and their composition determine the specific aroma of plants and the flavor of the resulting condiments. 

In general, the drying process leads to significant losses of volatile compounds. Among the drying treatments, some authors have reported higher losses of volatiles in convective hot air drying, and microwave drying as the air temperature and the wattage increased [72,91,92,93]. However, these statements were not observed in Hungarian thyme. In this particular case, as the air temperature increased, the losses decreased. This behavior was attributed to the longer processing at lower temperatures compared to the shorter times required for higher temperatures [88]. Volatile compounds retention was observed in studies on ginger when heat pump drying was applied. This is a positive effect with respect to other driers that usually loose the volatile compounds of the product [1,13]. 

Some authors have considered an initial hypothesis regarding the improvement produced by the microwave drying compared with traditional convective hot air drying. This improvement was reported by many authors in dried marjoram, thyme, oregano and basil [25,28,72,92,93]. However, other authors reported that rosemary and basil samples dried by convective drying showed higher yield of essential oils [91,94]. All these authors agree in the fact that the essential oil yield varies considerably from one species to another. The important losses of volatile compounds might be diminished by using assisted and combined drying techniques. For instance, Figiel [28] suggested that microwave drying assisted by vacuum increased the yield of essential oils in garlic, while others reported that the higher the vacuum intensity in the drying system for a specific microwave power and the higher the power intensity, the lower the concentration of total volatiles [28,95]. Besides, a convective pre-drying followed by microwave finishing drying increased the content of essential oils in samples of thyme, oregano, and rosemary [28,91,96]. Therefore, assisted techniques as vacuum-microwave drying and the combined techniques, such as convective pre-drying followed by vacuum-microwave finishing drying seem to be very promising options to dry aromatic herbs. 

Some authors have recently reported the effect of dehydration on the volatile compounds in different food products such as oyster mushrooms, shitake and chanterelle suggesting that the total concentration of volatiles of fresh mushrooms was drastically reduced by all drying treatments; although, the highest contents were found using (i) convective pre-drying (50 °C)-vacuum microwave finishing drying (480 W), and (ii) vacuum microwave drying at 480 W. Regarding dried shitake mushrooms the most recommended drying method in order to retain the highest content of volatile compounds was CD at 80 °C. Finally, for dried chanterelle the higher retention of volatile compounds content was registered when (i) convective drying at 80 °C, (ii) convective pre-drying followed by vacuum microwave finishing drying at 70 °C/480/240 W and (iii) convective pre-drying followed by vacuum microwave finishing drying at 80 °C/480/240 W were applied [97,98,99]. These new findings confirm that combined techniques tend to retain higher content of volatile compounds when compared to traditional (hot air convective drying) or modern techniques (vacuum-microwave drying) applied as a single treatment, improving the quality of some dried products and obtaining a reduction in the costs of the processing [28,29]. 

## 6. Sensory Properties of Dried Fruits, Vegetables, and Aromatic Herbs

Descriptive sensory analysis (DSA) is used to quantitatively determine the intensities of the main sensory properties and attributes of food. Such analysis requires the use of a trained panel. The proper number of panelists ranges between 7 and 12 of highly trained panelists [100]. DSA has been previously applied in the description of the main attributes of fruits, vegetables, but especially in aromatic herbs such as marjoram, thyme, basil, parsley, bay leaf, spearmint, and rosemary [76,91,92,95,96]. In general, dried samples of aromatic herbs have been typified by significant increases in the intensities of attributes such as spicy, hay-like, sweet, earthy, woody, and infusion. Modern drying techniques led to reduction of typical attributes of dried herbs. At the same time, an increase in the hot air temperature or microwave power led to decrease of some attributes such as fresh, floral and herbaceous [96]. 

Regarding DSA of dried fruits, some of the most affected attributes were sweetness, bitterness, adhesiveness, and caramel flavor. All these attributes are increased when the drying temperature and wattage also increased [72,96,101,102]. Recently, other authors applied osmotic dehydration as first steps during drying of pomegranate arils and highlighted that this technique with different solutions (pomegranate, chokeberry, and apple) provides samples without measurable off flavors. This finding justified that the solutions had high content of sugars (40%) and an attractive color [62]. 

**Figure 5:**
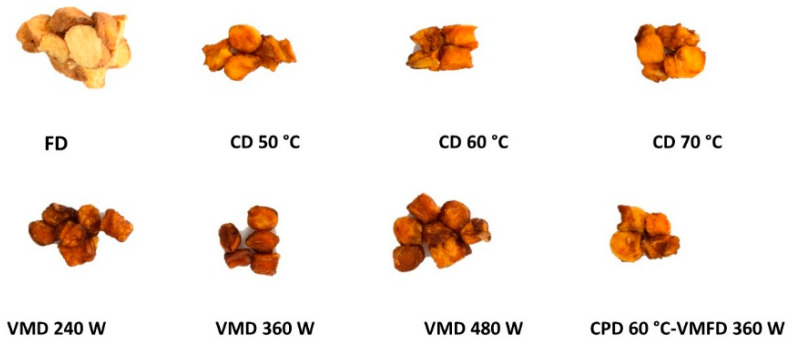
Effect of different drying techniques on sensory characteristics of loquat cultivar ‘*Algar*’ [102].

Product quality, consequently sensory properties, of dehydrated fruits is a key feature in innovation of future drying technology (Figure 5), which is closely related to: (i) development of novel drying techniques and (ii) process optimization [103]. Sensory evaluation will be a perfect tool to determine whether the effects of a particular drying technique will lead to high quality products, with elevated guarantee of being accepted by consumers. 

## 7. Conclusions

Research, development, and innovation are carried out in the food processing, especially in fruits, vegetables and aromatic herbs. Nowadays, consumers are demanding novel and healthier ready-to-eat products with long shelf-life and dehydrated products meet these criteria. Moreover, their functional properties and quality characteristics should be as close as possible to those of the fresh vegetal material. For these reasons, the food industry supports the research in both quality characteristics and processing techniques. In this sense, dehydration of agricultural products seems to be an extremely important matter in order to assure the physical, chemical and sensory quality of the final products. 

Recent studies concerning dehydration of agricultural products using vacuum-microwave technique showed that the most promising method is combined drying which ensures high quality at the lowest possible energy consumption. However, the optimization of novel drying techniques as well as the combination of them still requires additional studies in order to comprise other forms of treatments necessary to improve the texture, retention of valuable compounds (phytochemical and volatile compounds), health-promoting properties and an attractive sensory property. 

Finally, freeze-drying is one of the most recommended methods in terms of functional quality retention. However, from a physical point of view, the product can lose elasticity and become viscous when rehydrating. In order to avoid this issue, an osmotic pre-treatment is recommended. Thus, this phenomenon happens with many of the individual drying techniques, and for this reason, a combined technique is highly recommended depending on the kind of raw material. As it can be stated, all the techniques have strengths and weaknesses but the most important for a combined or novel technique is to reduce the costs, to be environmentally friendly, and to assure high quality of the dried products from a functional, physical, and sensorial point of view.
